# A Comprehensive Review of Innovative Paradigms in Microbial Detection and Antimicrobial Resistance: Beyond Traditional Cultural Methods

**DOI:** 10.7759/cureus.61476

**Published:** 2024-06-01

**Authors:** Shahzad Ahmad, Sham Lohiya, Amar Taksande, Revat J Meshram, Ashish Varma, Keta Vagha

**Affiliations:** 1 Pediatrics, Jawaharlal Nehru Medical College, Datta Meghe Institute of Higher Education & Research (Deemed to be University), Wardha, IND; 2 Pediatrics, Acharya Vinoba Bhave Rural Hospital, Wardha, IND

**Keywords:** nanotechnology, mass spectrometry, molecular-based techniques, innovative paradigms, antimicrobial resistance (amr), microbial detection

## Abstract

Microbial detection and antimicrobial resistance (AMR) surveillance are critical components of public health efforts to combat infectious diseases and preserve the efficacy of antimicrobial agents. While foundational in microbial identification, traditional cultural methods are often laborious, time-consuming, and limited in their ability to detect AMR markers. In response to these challenges, innovative paradigms have emerged, leveraging advances in molecular biology, genomics, proteomics, nanotechnology, and bioinformatics. This comprehensive review provides an overview of innovative approaches beyond traditional cultural methods for microbial detection and AMR surveillance. Molecular-based techniques such as polymerase chain reaction (PCR) and next-generation sequencing (NGS) offer enhanced sensitivity and specificity, enabling the rapid identification of microbial pathogens and AMR determinants. Mass spectrometry-based methods provide rapid and accurate detection of microbial biomarkers, including matrix-assisted laser desorption/ionization time-of-flight (MALDI-TOF) and biosensor technologies. Nanotechnology approaches, such as nanoparticle-based assays and nanopore sequencing, offer novel platforms for sensitive and label-free detection of pathogens and AMR markers. Embracing these innovative paradigms holds immense promise for improving disease diagnosis, antibiotic stewardship, and AMR containment efforts. However, challenges such as cost, standardization, and integration with existing healthcare systems must be addressed to realize the full potential of these technologies. By fostering interdisciplinary collaboration and innovation, we can strengthen our ability to detect, monitor, and combat AMR, safeguarding public health for generations.

## Introduction and background

Microbial detection plays a pivotal role in various fields including clinical diagnostics, food safety, environmental monitoring, and biodefense. Identifying and characterizing microorganisms accurately and rapidly is essential for effective disease management, infection control, and public health surveillance [1]. However, the emergence and spread of antimicrobial resistance (AMR) pose significant challenges to traditional microbial detection methods. AMR, fueled by the misuse and overuse of antibiotics, threatens the effectiveness of antimicrobial agents, leading to increased morbidity, mortality, and healthcare costs worldwide [2]. Traditional cultural methods, such as culture-based techniques and biochemical tests, have long been the cornerstone of microbial detection. While these methods have provided valuable insights into microbial pathogens, they are time-consuming and labor-intensive and often need more sensitivity and specificity, particularly in AMR surveillance [3]. Moreover, conventional approaches may fail to detect viable but non-culturable (VBNC) pathogens and polymicrobial infections, leading to diagnostic delays and inaccurate treatment decisions [4].

In response to the limitations of traditional cultural methods, there is a growing recognition of the urgent need for innovative paradigms in microbial detection and AMR surveillance [5]. Advances in molecular biology, genomics, proteomics, nanotechnology, and bioinformatics have paved the way for developing novel techniques that offer enhanced sensitivity, specificity, and rapidity in microbial identification and characterization. These innovative approaches hold immense promise for improving disease diagnosis, antibiotic stewardship, and AMR containment efforts [6]. This review article aims to provide a comprehensive overview of innovative paradigms in microbial detection and AMR surveillance, focusing on methods beyond traditional cultural approaches. By critically evaluating the strengths and limitations of emerging technologies, this review seeks to elucidate their potential applications in various settings, including clinical laboratories, public health agencies, and food production facilities. Additionally, this article highlights the challenges, opportunities, and future directions in microbial detection and AMR surveillance, underscoring the importance of embracing innovation to combat the global threat of AMR.

## Review

Traditional cultural methods for microbial detection

Overview of Traditional Techniques

Conventional methods for microbial detection, such as culture-based techniques and biochemical assays, have long been fundamental in microbiology. These methodologies entail the phenotypic identification of microorganisms through staining, culturing, and straightforward biochemical analyses. Culture-based methods typically involve the collection of samples, serial dilution, plating onto selective media, and awaiting an appropriate incubation period to observe visible colonies. This incubation period typically spans around 18-24 hours for most foodborne and waterborne bacterial pathogens. Quantifying bacteria in samples often relies on the enumeration of colony-forming units (CFUs) under controlled conditions, including specific parameters such as incubation temperature, duration, media selectivity, and oxygen levels [7,8]. Biochemical assays play a pivotal role in microbial identification by detecting specific enzyme systems or metabolic by-products within the culture medium. Common tests employed in microbiological laboratories for this purpose include the indole, methyl red, Voges-Proskauer, and citrate (IMViC) test, catalase test, oxidase test, urease test, and nitrate and nitrite reduction test. These assays furnish valuable insights to pinpoint target microorganisms based on their distinctive biochemical profiles [8].

Strengths and Limitations of Traditional Cultural Methods

The strengths of traditional cultural methods for microbial detection include their well-established structure and routine, efficient transmission of information, clear learning objectives, and direct face-to-face interaction between educators and students. These methods provide a structured framework that maintains a consistent routine in the classroom, offering a predictable learning environment. Lecture-based teaching allows for the efficient transmission of information, especially in subjects where foundational knowledge is crucial. Additionally, traditional teaching often has well-defined learning objectives, helping students understand expectations and educators cover topics systematically within a specified timeframe. Moreover, traditional education aligns well with standardized testing formats. It fosters direct interpersonal connections between educators and students, allowing for a deeper understanding of individual learning needs and creating a supportive environment [9]. However, traditional cultural methods also have several limitations. The culturing process can be lengthy and tedious, making it time-consuming and labor-intensive. These methods are also contamination-prone and require controlled conditions such as specific incubation times, temperatures, selective media, and oxygen availability. Traditional methods are less precise and efficient compared to more advanced technologies like next-generation sequencing (NGS) and whole genome sequencing (WGS). NGS and WGS offer more accurate and faster approaches to microbial detection and AMR, providing comprehensive insights that traditional methods cannot match [10,11].

Challenges in Detecting AMR Using Traditional Techniques

**Figure 1:**
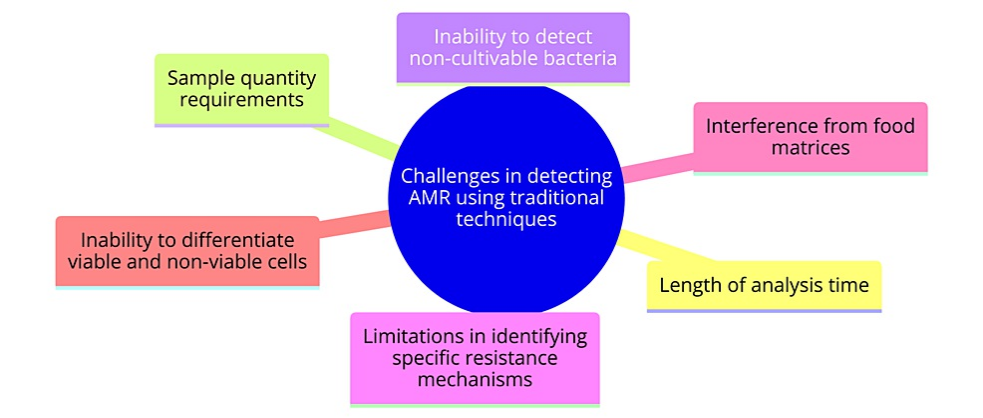
Challenges in detecting AMR using traditional techniques Image Credit: Dr. Shahzad Ahmad AMR: antimicrobial resistance

Traditional antimicrobial susceptibility testing (AST) methods, such as agar disk diffusion and broth microdilution, often require 18-24 hours or longer to yield results. This delay is too lengthy for timely clinical decision-making, where rapid intervention is crucial [12]. Furthermore, these methods demand a substantial quantity of the sample, posing challenges for certain clinical specimens where obtaining a sufficient amount can be difficult [12]. Another significant limitation is the inability of traditional culture-based techniques to detect non-cultivable bacteria. These methods can only identify bacteria that grow on the selected media, thus missing non-cultivable or slow-growing microorganisms that might carry AMR genes [13]. Additionally, while traditional phenotypic methods can reveal the presence of resistance, they often fail to identify specific resistance mechanisms or the exact genes responsible [13]. Food samples present further complications, as they may contain inhibitory substances that interfere with polymerase chain reaction (PCR)-based detection of AMR genes when using traditional methods [13]. Moreover, conventional PCR techniques cannot differentiate between viable and non-viable cells, detecting resistance genes in both. This limitation makes it challenging to ascertain the true resistance profile of the sample [13]. Challenges in detecting AMR using traditional techniques are shown in Figure 1.

Innovative paradigms in microbial detection

Molecular-Based Techniques

PCR: Molecular-based techniques, especially PCR, have revolutionized molecular biology by enabling the rapid and precise amplification of specific DNA sequences [14,15]. PCR uses short synthetic DNA fragments called primers to target and amplify a particular segment of the genome, followed by repeated cycles of DNA synthesis to amplify that segment [14]. Developed in 1983 by American biochemist Kary Mullis, PCR has become an indispensable tool in molecular biology and biotechnology [14]. The PCR process comprises three main cyclic reactions: denaturation, annealing, and extension. During denaturation, the reaction mixture is heated to 94°C for 0.5-2 minutes, breaking the hydrogen bonds between the DNA strands to create single-stranded DNA [14]. The temperature is then lowered to 54-60°C for 20-40 seconds, allowing the primers to bind to their complementary sequences on the template DNA [14]. In the extension step, the Taq polymerase enzyme adds bases to the 3’ end of the primers, elongating the DNA in the 5’ to 3’ direction [14]. PCR has numerous applications, including detecting genetic mutations, monitoring gene expression, identifying disease-causing genes, and genetic fingerprinting [14]. It is also essential in gene mapping, phylogenetic analysis, and gene expression studies [14]. The technique's high sensitivity allows it to amplify minimal DNA samples, making it a powerful tool in forensic science and medical diagnostics [14]. Moreover, PCR has led to the development of various PCR-based techniques, such as real-time PCR, digital PCR, and inverse PCR, each offering specific advantages and expanding PCR's capabilities in DNA sequence analysis with greater precision and efficiency [16].

NGS: NGS is a groundbreaking technology that transforms genetic analysis by enabling the rapid and precise sequencing of DNA and RNA. NGS can sequence hundreds to thousands of genes or even entire genomes quickly, offering significant advantages over traditional Sanger sequencing methods [17,18]. The process involves several key steps, including DNA fragmentation, library preparation, massive parallel sequencing, bioinformatics analysis, and annotating and interpreting variants and mutations [17]. NGS has a broad range of clinical applications, from disease diagnosis and prognosis to guiding therapeutic decisions and patient follow-up, making it an essential tool in personalized precision medicine [17]. The technology's scalability, speed, and ability to detect variants at lower allele frequencies have revolutionized genetic analysis, enabling new applications in genomic and clinical research, reproductive health, and other scientific fields [18]. NGS data analysis comprises primary, secondary, and tertiary steps, with bioinformatics specialists playing a crucial role in interpreting results and extracting meaningful information from the data [19].

Microarray technology: Microarray technology, a sophisticated molecular-based technique, enables the comprehensive genetic analysis of various organisms for diagnosing bacterial, viral, fungal, and parasitic diseases at the genus and species levels. Microarray technology involves attaching known nucleic acid fragments to a solid surface, creating a "chip" that interacts with DNA or RNA from a sample to produce detectable fluorescence through complementary base pairing [20]. This technology is crucial for measuring gene expression, detecting specific DNA sequences like single-nucleotide polymorphisms (SNPs), and conducting genome-wide association studies (GWASs) [20,21]. Microarrays effectively detect large chromosomal abnormalities, copy number variations (CNVs), and SNPs, making them invaluable for gene expression profiling, cancer diagnostics, Mendelian disease studies, and reproductive health assessments [21]. Despite their reliance on specific probes for detection, microarrays remain a key diagnostic tool in clinical laboratories due to their ability to probe a vast number of genes simultaneously and their high-resolution capabilities [21]. In the short-to-medium term, microarrays are expected to continue expanding in genetic testing applications due to their efficiency in analyzing multiple genome areas simultaneously [21].

Mass Spectrometry (MS)-Based Methods

Matrix-assisted laser desorption/ionization time-of-flight (MALDI-TOF): MALDI-TOF MS is a highly advanced analytical technique that ionizes samples into charged molecules, facilitating the measurement of their mass-to-charge ratio (m/z) [22,23]. In MALDI-TOF MS, the ion source is MALDI, and the mass analyzer is a TOF analyzer. This technology is advantageous for analyzing biomolecules like peptides, lipids, saccharides, and other organic macromolecules without causing fragmentation or decomposition. It is suitable for large biomolecules that might degrade under traditional methods [22]. MALDI-TOF MS is essential for rapidly detecting oligonucleotide fragments, ensuring synthesis completeness and sequence accuracy. It is renowned for its simplicity, speed, accuracy, and sensitivity in determining oligonucleotide sequences [22]. Additionally, MALDI-TOF MS can be used for MALDI imaging mass spectrometry (MALDI-IMS), which enables the direct profiling and imaging of proteins from tissue sections. This provides detailed information on the molecular composition, abundance, and spatial distribution of peptides and proteins within tissues [22]. The technology is versatile and capable of analyzing a broad range of biomolecules with low internal energy ions, resulting in minimal fragmentation and easy maintenance with fast data acquisition [22]. Selecting the appropriate matrix substance is crucial for successful MALDI-TOF MS, ensuring the solubility of the analyte and the matrix in specific solvents for optimal results [22].

Electrospray ionization MS (ESI-MS): MS-based methods, particularly ESI-MS, have transformed analytical chemistry by providing a powerful tool for analyzing molecular species and their association/dissociation [24,25]. ESI-MS is a gas-phase method that ionizes macromolecules using a "soft" ionization process, which preserves the integrity of the molecules, allowing for the detection of intact molecular species [26]. This technique is particularly beneficial for analyzing biological macromolecules such as proteins, peptides, and oligosaccharides, as it overcomes the tendency of these molecules to fragment when ionized [27]. The ability to detect very low levels of analytes (femto- and attomole levels) and the flexibility offered by coupling ESI with various mass analyzers and chromatographic techniques make ESI-MS a versatile tool for a wide range of applications [27]. Advantages of ESI-MS include the ability to study biological macromolecules in their native state, retain solution-phase information in the gas phase, and analyze large biomolecules such as membrane proteins [27]. The technique's significance in analyzing biological macromolecules was recognized when John Bennett Fenn and Koichi Tanaka were awarded the Nobel Prize in Chemistry in 2002 for developing ESI for analyzing biological macromolecules [27]. ESI-MS has been applied to numerous fields, including protein folding studies, lipid analysis, and noncovalent gas-phase interaction studies [27,28]. Additionally, the technique has facilitated new strategies for MS-based lipid analyses, including shotgun lipidomics, which involves directly infusing crude biological extracts into the mass spectrometer. This ability to analyze lipids directly from biological samples has proven to be a powerful tool for understanding lipid metabolism and its role in disease [27,28].

Biosensor Technologies

Overview of biosensors: Biosensors are pivotal analytical devices integrating a biological sensing element with a physical transducer to detect chemical substances. They have significant applications across various fields, including biomedical diagnosis, environmental monitoring, food safety, and drug discovery. The biological element within a biosensor interacts with the analyte, producing a signal that the transducer converts into a measurable and quantifiable output [29]. Key components of a biosensor include the analyte (the substance of interest), bioreceptor (the molecule that recognizes the analyte), transducer (which converts energy forms), electronics (for signal processing), and display (for presenting results). Bioreceptors can be enzymes, cells, aptamers, DNA, or antibodies. The transducer typically converts the bio-recognition event into an optical or electrical signal proportional to the analyte-bioreceptor interaction. The electronics then process and quantify these signals for user-friendly display [30]. With advancements in nanotechnology, biosensors have evolved into highly sensitive and miniaturized devices, crucial for rapid, cost-effective analysis in disease diagnosis, medicine, and environmental monitoring. The continuous progress in biosensor technology includes integrating various biosensor types and transduction techniques to enhance detection capabilities and address emerging challenges [31].

Application in microbial detection and AMR surveillance: Biosensors, particularly microbial biosensors, detect target analytes by immobilizing microorganisms onto transducers. These devices facilitate real-time microbial activity and antibiotic response monitoring, enabling timely interventions and personalized treatment strategies [32]. In AMR surveillance, biosensors are employed for the phenotypic and genotypic detection of resistance mechanisms. Phenotypic techniques assess the expression of resistance traits, while genotypic tools identify specific genetic markers associated with resistance. Biosensors provide a rapid, cost-effective, and sensitive means of detecting resistance genes and assessing bacterial susceptibility to antibiotics, guiding appropriate treatment decisions, and combating the spread of resistant strains [33,34]. Integrating biosensor technologies with advanced methods such as surface-enhanced Raman spectroscopy (SERS) and artificial intelligence enhances the accuracy and efficiency of microbial detection and AMR surveillance. These approaches enable high-throughput screening, multiplexed analysis, and real-time data collection, revolutionizing the field of infectious disease management and antibiotic stewardship [35].

Nanotechnology approaches: Nanoparticle-based assays are cutting-edge tools that exploit the unique properties of nanoparticles for various applications, including bioaffinity assays, immunoassays, and point-of-care testing (POCT). These assays employ nanoparticle labels for detection, signal generation, and amplification, offering enhanced sensitivity and specificity in identifying target molecules [36]. In bioaffinity assays, nanoparticles have shown great potential to replace traditional enzymatic or molecular labels used in immunoassays. Notably, they have been successfully integrated into commercial assays, such as lateral flow assays, where rapid antigen tests for severe acute respiratory syndrome coronavirus 2 (SARS-CoV-2) have exemplified the visual detection of red-colored lines formed by colloidal gold nanoparticles. These tests have quickly transitioned from initial laboratory research to widespread market adoption, highlighting the efficacy of nanoparticle-based assays for rapid and effective diagnostics [36]. Nanoparticle-based immunochemical biosensors and assays have been evaluated using digital methods, enabling single-molecule analysis through counting individual molecular or nanoparticle interactions. This digital evaluation significantly enhances the sensitivity and accuracy of immunoassays, providing a more precise and reliable detection method [37]. Gold nanoparticle-mediated lateral flow assays have also been developed for detecting host antibodies and coronavirus disease 2019 (COVID-19) proteins. These assays utilize gold nanoparticles as signal reporters, with the nanoparticles' size and shape influencing the assay's sensitivity and detection limit. Techniques such as silver enhancement, galvanic replacement, and gold enhancement have been employed to boost these assays' sensitivity, demonstrating nanoparticle-based detection systems' versatility and adaptability [38].

Advantages of innovative paradigms

Enhanced Sensitivity and Specificity

The integration of PCR followed by ESI-MS with an efficient lysis method and automated DNA purification system has substantially improved the sensitivity of bacterial detection in blood specimens. This advancement has resulted in higher detection rates than traditional culture methods [39]. Phage-modified magnetoplasmonic nanoparticles have demonstrated remarkable specificity in detecting specific bacterial strains, such as *Staphylococcus aureus*, even in the presence of other bacterial species. This technology showcases the high specificity achieved through its application [40]. SERS stands out as a powerful technique for bacterial detection, offering rapid, sensitive, and non-destructive capabilities. It provides molecular fingerprint information and facilitates the online qualitative analysis of multicomponent samples, underscoring its effectiveness in enhancing sensitivity and specificity in bacterial detection [41]. The development of a nano-biosensor based on iron nanoparticles has demonstrated enhanced sensitivity and efficiency in detecting *S. aureus*. This innovation addresses the limitations of traditional methods by offering improved sensitivity and specificity in bacterial detection [42].

Rapid Detection and Identification of Microbes

Rapid microbial methods (RMMs), also referred to as alternative microbiological methods, offer quicker microbiology test outcomes compared to traditional culture-plate techniques. These methods yield results within hours, as opposed to the days or weeks required by conventional approaches, and can be categorized into qualitative, quantitative, and identification methods [43]. Technological advancements have been instrumental in the evolution of rapid microbial detection methods. Nucleic acid amplification methods, such as PCR and fluorescence-based detection methods, have been found to be applicable in industrial rapid microbiology. These techniques amplify and detect nucleic acids within microbial cells or employ fluorescence to detect bacterial cells, necessitating a brief growth-based pre-enrichment for achieving an acceptable detection limit. Emerging methodologies like solid-phase and flow cytometry represent nongrowth-based approaches with the potential for heightened sensitivity compared to earlier rapid methods. Despite certain drawbacks, these techniques exhibit promise in microbial detection and identification [44]. The use of rapid microbial detection and identification methods extends across various domains, including healthcare and food safety. In healthcare settings, rapid methods are being employed for the swift detection and identification of microorganisms from blood cultures, presenting a more efficient alternative to traditional approaches that involve Gram's staining of blood culture aliquots. This accelerated detection capability significantly impacts patient care, enabling timely and targeted treatment interventions. In the food industry, rapidly detecting and identifying microbes are paramount for ensuring food safety. Projects are underway to assess molecular probes for the specific enumeration of bacteria using direct epifluorescence microscopy. This technology can revolutionize food safety monitoring by furnishing rapid and precise results, facilitating prompt intervention in the event of contamination [1].

Capability to Detect AMR Markers Directly

NGS is a potent tool that detects genomic changes in microorganisms, particularly those linked to AMR. This technology furnishes high-throughput capabilities, facilitating the identification of low-frequency variants and genomic arrangements associated with AMR [45,46]. Spectroscopic techniques, such as nuclear magnetic resonance (NMR) and Fourier-transform infrared (FTIR) spectroscopy, offer avenues for scrutinizing molecules at the atomic level, thereby providing invaluable insights into AMR mechanisms. These methods allow conducting multiple studies on the same sample, directly detecting AMR markers [12]. Molecular-based assays, encompassing WGS and whole metagenome sequencing (WMS), emerge as effective means for directly identifying AMR genes and mechanisms. These assays offer high-resolution variant detection without necessitating specific probes, thus enhancing the detection of AMR markers [45].

Potential for POCT

POCT presents several advantages that significantly impact healthcare delivery. Firstly, its rapid turnaround time expedites diagnosis and treatment access, reducing patients' time in hospitals and emergency departments [47,48]. Secondly, POCT enhances the efficiency of the care process by diminishing referrals for additional tests, shortening the length of stay in healthcare facilities, and decreasing the likelihood of admissions [47,48]. Moreover, POCT contributes to cost-effectiveness by streamlining the diagnostic process and treatment decisions, ultimately reducing the overall cost of care and aligning with efficiency targets while optimizing resource allocation [47,48]. Furthermore, POCT facilitates improved clinical decision-making by enabling immediate action based on test results, thereby facilitating timely treatment interventions and mitigating the risk of complications [47,48]. Modern POCT devices offer expanded test menus and connectivity, consolidating a wide range of tests onto a single platform. This consolidation enhances efficiency and reduces turnaround time. Additionally, these devices are increasingly integrated with electronic health records, facilitating real-time data sharing and comprehensive patient monitoring [47,48]. Lastly, advancements in technology have further elevated the capabilities of POCT devices. Integration of advanced technologies such as the Internet of Things, artificial intelligence, and biosensors enhances the accuracy, effectiveness, and reliability of POCT devices. These advancements contribute to improved patient care and treatment outcomes, solidifying the role of POCT as a pivotal component of modern healthcare delivery [47,48].

Automation and High-Throughput Capabilities

Automated systems offer numerous benefits that revolutionize microbial screening and testing processes. Firstly, they significantly enhance efficiency by concurrently handling many parallel experiments, accelerating the screening and testing processes [49,50]. Secondly, through high-throughput screening (HTS) techniques, automated systems enable the miniaturization of experiments, reducing sample and reagent volumes while preserving accuracy and reproducibility [49,50]. Additionally, these systems provide substantial time savings compared to traditional manual methods. For instance, commercially available molecular detection systems can deliver results within 1-2 hours, a stark contrast to the 18-24 hours typically required by culture-based methods [49,50]. Moreover, automated systems are designed with user-friendliness in mind, requiring minimal training for operation. This characteristic makes them accessible to a broader range of users across various settings [49,50]. Furthermore, studies have demonstrated the potential for scale-up with automated microbial bioreactor systems like the ambr 15f. These systems facilitate strain and molecule selection and rapid scale-up, showcasing a remarkable potential for 1,300-fold scale comparability in both growth and product yield [50]. Lastly, automated systems contribute to improved accuracy by eliminating human errors and biases, thereby providing more consistent and reliable results, particularly in AST [51]. Overall, adopting automated systems represents a significant advancement in microbial screening and testing, offering increased efficiency, time savings, ease of use, scalability, and improved accuracy.

## Conclusions

This review has highlighted the pressing need to move beyond traditional cultural methods in microbial detection and AMR surveillance. We've delineated conventional approaches' shortcomings and underscored innovative paradigms' transformative potential. By harnessing molecular techniques, MS, biosensors, and nanotechnology, we can achieve heightened sensitivity, specificity, and speed in identifying and characterizing microbial pathogens. Embracing these innovative methods is paramount in combating the escalating threat of AMR, as they enable targeted antimicrobial therapy, proactive surveillance, and infection control measures. Looking ahead, the future of microbial detection and AMR surveillance holds promise for further advancements, driven by the integration of artificial intelligence, miniaturization of diagnostic platforms, and interdisciplinary collaboration. As we navigate the evolving landscape of AMR, adopting innovative solutions will be pivotal in safeguarding public health and preserving the efficacy of antimicrobial agents worldwide.
